# Study of the Technical Feasibility of Increasing the Amount of Recycled Concrete Waste Used in Ready-Mix Concrete Production

**DOI:** 10.3390/ma10070817

**Published:** 2017-07-18

**Authors:** Esteban Fraile-Garcia, Javier Ferreiro-Cabello, Luis M. López-Ochoa, Luis M. López-González

**Affiliations:** 1 SCoDIP Group, Department of Mechanical Engineering, University of La Rioja, 26004 Logroño, Spain; javier.ferreiro@unirioja.es; 2 GI-TENECO Group, Department of Mechanical Engineering, University of La Rioja, 26004 Logroño, Spain; luis-maria.lopezo@unirioja.es (L.M.L.-O.); luis-maria.lopez@unirioja.es (L.M.L.-G.)

**Keywords:** concrete, ready-mixed concrete, aggregate, recycled concrete waste, recycled

## Abstract

The construction industry generates a considerable amount of waste. Faced with this undesirable situation, the ready-mix concrete sector, in particular, has invested energy and resources into reusing its own waste in its production process as it works towards the goal of more sustainable construction. This study examines the feasibility of incorporating two types of concrete waste, which currently end up in landfill, into the production process of ready-mix concrete: the waste generated during the initial production stage (ready-mix concrete waste), and waste created when demolition waste is treated to obtain artificial aggregate. The first phase of the study’s methodology corroborates the suitability of the recycled aggregate through characterization tests. After this phase, the impact of incorporating different percentages of recycled coarse aggregate is evaluated by examining the performance of the produced concrete. The replacement rate varied between 15% and 50%. The results indicate that recycled aggregates are, indeed, suitable to be incorporated into ready-mix concrete production. The impact on the final product’s performance is different for the two cases examined herein. Incorporating aggregates from generic concrete blocks led to a 20% decrease in the produced concrete’s strength performance. On the other hand, using recycled aggregates made from the demolition waste led to a smaller decrease in the concrete’s performance: about 8%. The results indicate that with adequate management and prior treatment, the waste from these plants can be re-incorporated into their production processes. If concrete waste is re-used, concrete production, in general, becomes more sustainable for two reasons: less waste ends up as landfill and the consumption of natural aggregates is also reduced.

## 1. Introduction

As more buildings are demolished every day, effectively reusing demolition waste is essential to conserving non-renewable natural resources [1,2,3,4]. The construction industry, in general, and concrete sector, in particular, are under considerable pressure to reduce their environmental impact. Thus, the construction community has made an effort to reduce its consumption of extracted natural resources and to establish sustainable alternatives to manage construction and demolition waste as well [5]. Over the last two decades, a variety of recycling methods for construction and demolition wastes (CDW) have been developed. For instance, one of the major components in CDW, concrete rubble, has been used to replace natural aggregate after being treated. This material is known as recycled concrete aggregate (RCA) [6]. Despite the production of CDW aggregate, the quantity of waste generated has not subsided. The volume of concrete demolition waste is increasing primarily because many old buildings and other structures have surpassed their limit of use and need to be demolished [7].

It is important to remember that the source of waste conditions its subsequent reuse. Nevertheless, there is often a lack of information available regarding the origins of waste. For example, using waste from different sources does not represent a problem in the case of subbases for transportation infrastructure [8,9,10,11]. A common source of aggregate are recycling centers for construction waste. These centers contain rubble from various sources and, therefore, the properties of the aggregate are not uniform [12,13]. Another source of aggregate can also be precast concrete or brick plants. In such plants, various products are normally made from the same type of concrete, so variability in the properties of the rubble should not be an issue [12,14]. Thus, recycled concrete used to make coarse aggregate for new concrete can be obtained from the demolition of concrete elements in roads, bridges, buildings, and other structures, or fresh and hardened rejected units in precast concrete plants [15].

RCA was originally used as landfill and mainly for low-value purposes, because of limited recycling facilities and for economic efficiency. Nowadays, this situation has changed after extensive research and because of recent significant advances in modern sustainable concrete technology. The first applications of RCA were implemented in civil engineering [16]. Research has revealed that RCA’s relevant material properties are generally inferior compared to those of conventional concrete, but that RCA is still adequate for practical applications in civil engineering [17,18]. At present, RCA is widely used for non-structural concrete applications, such as coarse materials for road bases, paving blocks, and embankment fills [6,19,20]. In fact, RCA has also been incorporated in applications that would allow the maximum size of aggregate to be increased [21]. Other applications of RCA have focused on using it as an ingredient in structural concrete [22,23,24]. For this type of concrete, production processes involving demolition waste and ready-mix concrete waste both require that CDW undergo prior treatment.

Clearly, concrete made with recycled aggregates is no longer merely a research topic but, rather, it is already a practical reality. For several years, a number of countries have been pioneering research in this area [25]. Various pilot projects have been implemented with encouraging results [2,25,26]. The use of RCA is so widespread in certain regions that some countries have already developed, or are currently in the process of developing, regulations regarding its use and characteristics [25]. The majority of regulatory codes restrict or even prohibit the use of fine recycled aggregates in concrete production because of their inadequate properties [25,26,27]. However, some studies suggest that their use is not necessarily inauspicious and that satisfactory results (similar to those obtained with fine natural aggregates) are feasible in concrete which contains a proportion of this type of aggregate [25,26,28]. Thus, the use of RCA for actual structures may be limited, but it is not prohibited. Accordingly, European Standard EN 12620 [29] has already specified requirements for recycled aggregates so that they may be used in the production of structural concrete. Several countries have already drafted national regulations that define the conditions under which these types of aggregates can be utilized [5,30,31,32,33,34].

Several different teams of researchers have obtained results [1,15,20,35,36,37,38,39] that demonstrate that RCA can be adequate for structural purposes. However, concrete made with 100% coarse recycled aggregate requires a large amount of cement to achieve high compressive strength. Hence, this is not a viable economical alternative, as it is not cost effective [40,41]. It is interesting to note that using concrete with 100% recycled coarse aggregate for lower grade applications is allowed in Hong Kong, though for higher grade applications (above M35 concrete), only 20% replacement is permitted [42]. This issue is more pronounced in the case of fine recycled aggregates, as opposed to coarse recycled aggregates [40]. Incorporating fine recycled aggregates in high-strength concrete requires additional reinforcement [43,44,45]. Furthermore, since recycled concrete aggregate is always collected from different sources or types of concrete, the basic engineering properties, such as shape and texture, specific gravity, absorption, moisture content, permeability, strength characteristics, deleterious substances, resistance to freeze–thaw, etc., vary considerably. As a result, significant variations in the engineering properties of concrete made with recycled concrete aggregate have been reported [45,46,47]. In Japan, JIS has drafted a technical report, TRA 0006 “Recycled Concrete Using Recycled Aggregate” to promote the use of concrete made with recycled aggregate. Relevant standards for recycled materials would provide producers with targets and users with assurance regarding the quality of the material [42]. Another interesting study is Katz’s classification [12] which highlights one of the major difficulties in recycling demolition waste: the variable and unpredictable quality of recycled aggregates. Among the different types of RCA, coarse recycled aggregates are the most promising option. This study evaluates two sources of coarse recycled aggregate in M25 concrete production for building structures.

The European Commission emphasizes that converting waste into a resource is key to improving the efficient use of resources and advancing towards a more circular economy. In areas where natural resources are scarce, the feasibility of recycled concrete waste has been confirmed [42]. In areas where natural resources are abundant, environmental feasibility is compromised when more cement is used in mixes and transportation [48,49,50]. The European Commission’s objectives include: reducing landfill dumping to 10% or less of total waste by 2030, and intensify collaboration between member States to improve the management of landfill waste. The financial viability of these objectives must not be overlooked [51]. In Spain, the first and second national plans for CDW were implemented with the goal of reusing construction waste in concrete production [52]. At present, in Spain, several research groups are working together to develop specific regulations for the use of recycled concrete materials in concrete production [53,54]. Etxeberria et al. [40] affirm that to obtain high-quality concrete using recycled aggregate, the minimum requirements defined by DIN 4226 [30] and UNE EN13242:2003 + A1:2008 [55] must be fulfilled.

The key to taking advantage of and re-using waste is accurately characterizing it, and then determining the amount of recycled aggregate that can be incorporated into an RCA mix. This task should be undertaken by the entity generating said waste, thus avoiding heterogeneous mixes and unnecessary operations.

The present study focuses on proving the feasibility of incorporating recycled aggregate, generated during concrete production, into new structural concrete. The strength properties of the concrete obtained dictate its future use or application. By comparing concrete waste, from two sources in the construction sector, the new concrete can be evaluated.

In order to reach this goal, homogenous recycled aggregates must be used that are created and managed during the production process at a ready-mix concrete plant. Improvements in production processes ought to aim for the lofty goal of zero waste. The two cases presented herein constitute examples that may serve as an incentive for other ready-mix concrete plants or even precast concrete companies that could recycle their own waste. The party generating the waste should implement guidelines and procedures for reusing waste.

The source of the material to be recycled affects the properties of the concrete made with those recycled materials. The concrete sector should re-use waste generated during production and from concrete structure demolition.

In this study, two sources were chosen: ready-mix concrete waste from the company Hormigones Ebro and demolition waste from Hormigones Rioja. These two types of waste were analyzed and compared to characterize the concrete made with the recycled materials. Given the results of this study, the construction sector should consider using recycled materials in a variety of applications.

## 2. Methodology

This study analysed the behaviour of different types of concrete made with recycled aggregates in terms of their mechanical properties. The final objective was to evaluate the possibility of re-using waste and thereby contribute to a closed cycle in the construction industry. Coarse aggregate was partially replaced in different percentages and then, the new concrete’s properties were compared with the reference concrete. The driving idea is to promote the utilization of recycled aggregate in the construction industry in order to reduce landfill waste and reign in the unfettered use of natural resources, with the end goal of consolidated sustainable development in the construction sector. Circular economy theory and practice were implemented at two ready-mix concrete plants in Spain applying the corresponding regulations.

In this endeavour, the properties of the different concrete compositions were analysed wherein five different percentages of recycled aggregate replaced coarse aggregate: 0%, 15%, 20%, 30%, and 50%. Concrete properties were evaluated in fresh and hardened states.

**Figure 1:**
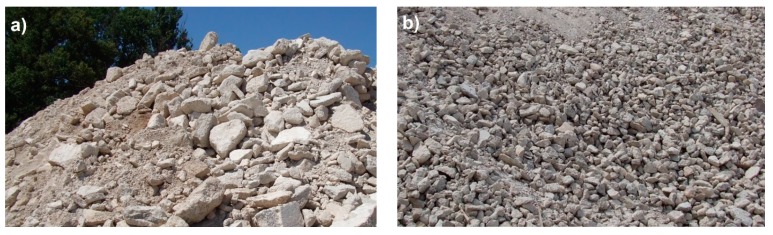
(**a**) Concrete waste. Source: Hormigones Ebro; and (**b**) demolition waste. Source: Hormigones Rioja.

Natural aggregates (NA), gravel, and sand, produced by crushing and supplied by La Peña quarry, were incorporated into concrete production in three different percentages. These materials are of siliceous origin. The company Hormigones Ebro normally utilizes them to manufacture concrete. The recycled aggregate (RA) came from two different sources:

- Hormigones Ebro (HE). Aggregate made from the waste of concrete blocks that were not classified beforehand (Figure 1a).
- Hormigones Rioja (HR). These aggregates come from the rejection stage of the industrial process for recycling demolition materials to obtain artificial aggregate.

**Figure 2:**
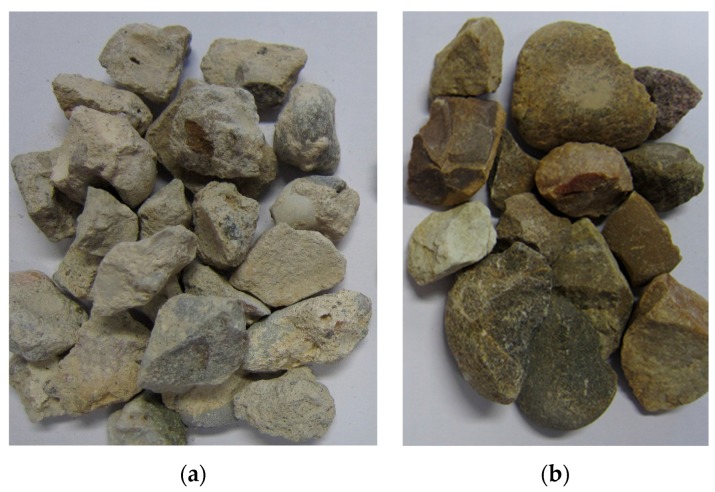
Coarse aggregate utilized: (**a**) derived from concrete waste; and (**b**) natural aggregate.

Both images depict aggregates derived from concrete, but there are some differences between the two types. HE recycled aggregate is waste from different types of concrete (primarily concrete used for building construction HA-25-B-20-IIa) that it crushed and sieved once. On the other hand, the HR recycled aggregate derived from demolition concrete is used to obtain artificial aggregate after undergoing treatment. This process involves eliminating large pieces of concrete. This material is then crushed and classified to be used as recycled aggregate. In both plants, CA-12/20-T-R aggregate was obtained after crushing and then sieving. Figure 2 displays the two aggregates obtained.

The different physical and chemical properties of these recycled aggregates were assessed by the tests detailed below. The first step is to evaluate the aggregates’ suitability for structural concrete. Then the specific materials and the different percentages of aggregate to be utilized in concrete manufacturing are defined. The methodology employed to manufacture concrete in the laboratory is also presented, as well as the methodology followed in the characterization tests conducted on the different concretes produced.

### 2.1. Characterization of Recycled Aggregate

Before utilizing recycled aggregates in structural concrete, their suitability must be confirmed by means of the corresponding tests. A description of the proposed testing methods to characterize recycled coarse aggregate (12/20) is included below, including the limit values stipulated by regulations. These requirements are divided into two sections: physical-mechanical requirements and chemical requirements.

#### 2.1.1. Physical Properties of RCA

In order to guarantee the fulfilment of the physical-mechanical requirements, certain tests must be conducted. The most relevant aspects are included below and the tests carried out as well.

The size and shape of the aggregates to be incorporated into structural concrete affect the strength performance. The geometric properties of aggregates were determined by the sieving method indicated in regulation UNE-EN 933-1:2012 [56]. In addition to the requirements regarding natural aggregate, Annex 15 of EHE 08 [34] establishes that the percentage of eliminated small particles in recycled aggregates cannot be greater than 10%, and the particle content that passes through the 4 mm sieve must not be greater than 5%. The amount of fine particles permitted is 1.5% of the coarse aggregate amount. If there is an excess of fine particles on the aggregate surface, adherence between the aggregate and the mortar decreases and, furthermore, fine particles also lead to an increase in the quantity of water in the concrete mix.

The presence of clay in the coarse aggregate fraction is also subject to restrictions, especially in the case of those aggregates made from recycled materials. For concrete wherein recycled aggregate constitutes no more than 20% of the total aggregate quantity, the amount of clay lumps must not be greater than 0.6%, and the clay content in the fraction of natural coarse aggregate cannot exceed 0.15%. In the extreme case of using 100% recycled coarse aggregate, the percentage of clay lumps in the aggregate must not exceed 0.25% [34]. When working with aggregate from demolitions, these undesirable materials are found quite often and create problems for producing quality concrete.

To produce quality concrete, the correct amount of water is essential, as indicated by regulations regarding the water to cement ratio. Therefore, it is imperative that one be familiar with the water absorption of the different components of concrete. This value is determined for recycled aggregates according to regulation UNE-EN 1097-6:2014 [57]. To manufacture concrete with a recycled aggregate content no higher than 20%, the absorption rate must not exceed 7%. In addition, natural coarse aggregate must not have an absorption rate higher than 4.5%. For concrete produced with more than 20% recycled aggregate, the combination of natural and recycled coarse aggregate must comply with regulation EHE-08, with an absorption coefficient no greater than 5%. Absorption rates are normally greater in recycled aggregate due to the quantity of adhered mortar that such aggregates contain. This absorption rate indicates the quantity of water the concrete needs in order to adequately hydrate the cement. In the case of aggregate’s wear resistance, regulations do not distinguish between different sources for coarse aggregates. The Los Angeles coefficient value must not exceed 40%. The Los Angeles coefficient for recycled aggregates presents higher values due to the weight loss that occurs in natural aggregate during testing, and also as a result of completely eliminating the adhered mortar. Regulation UNE-EN 1097-2:2010 [58] stipulates how to conduct testing to determine resistance to fragmentation.

Resistance to freezing and thawing cycles is related to the strength of aggregate particles and the distribution of pores in the aggregate. Recycled aggregates lose more weight than natural aggregates, because of the mortar adhered to the rock matrix when subjected to 10 frost/thaw cycles in water or five cycles in a magnesium sulphate solution. In order to determine the maximum weight loss experienced by recycled aggregates when subjected to treatment cycles with sulphate magnesium solutions, the sample must undergo prior preparation: consisting of thorough washing and sieving with a 10 mm sieve, to eliminate all the friable particles (which crumble easily) before proceeding with the test outlined in regulation UNE-EN 1367-2:2010 [59]. The EHE establishes the limit for the test result at 18%, which is the same requirement set for natural aggregate.

#### 2.1.2. Chemical Properties of RCA

There is a second set of requirements for aggregates involving chemical testing. These tests are necessary to guarantee the quality of the concrete made from the different types of aggregate. Chlorides in concrete must be limited when dealing with reinforced structural concrete. In this type of concrete, chlorides must be within permissible values so as to avoid corrosion of reinforcement. Chloride content present in recycled aggregates is an important factor to consider when aggregates come from construction sites where they may have come into contact with liquid salts, such as in high mountainous areas, or in direct contact with seawater, or if during the manufacture of concrete some accelerating product was employed. Being aware of the source of waste allows for more efficient management. Nevertheless, even if the source is known, in the case of recycled aggregates, it is also advantageous to determine the quantity of soluble chlorides in water and the total amount of chloride contained in the aggregate. This is significant information because of the fact that different combinations of chlorides may exist and can be reactive, such as the case of the chlorine hydrated calcium aluminate, which can release chloride ions in the presence of sulphate ions. EHE-08 limits the maximum content of soluble chlorides in water to 0.05% for coarse and fine aggregate when utilized for reinforced concrete. If aggregates are to be used to manufacture pre-stressed concrete, said limit is reduced to 0.03%. This percentage applies to the mass of dry aggregate (the same specification used for total chlorides) and is addressed in Article 7 of regulation UNE-EN 1744-1:2010 [60].

The amount of sulphates must also be controlled in materials to be used in concrete production. The quantity of sulphate contained in recycled aggregate can be a significant factor due to the sum of sulphate corresponding to natural aggregates and that of aggregate’s adhered mortar. In the case of concrete from building demolitions, gypsum is also considered a contaminant. These sulphates can cause problems in concrete since they can combine with hydrated tricalcium aluminate in cement to create ettringita (hydrated tricalcium sulphoaluminate) and provoke detrimental expansions. Another possible transformation is that of free calcium hydroxide, or calcium released during concrete hydration, which also entails an increase in volume that can fracture concrete. The content of soluble sulphates in acid, expressed as SO_3_ in coarse and fine aggregates, is determined according to Article 12 of regulation UNE-EN 1744-1:2010 [60], and cannot exceed 0.8% of the aggregate mass. In order to reduce the risk of expansive chemical reactions in concrete as much as possible, the total content of sulphur compounds is limited, as opposed to restricting just water-soluble. The goal is to considerably lower the risk of expansion. Thus, by reducing the amount of gypsum in recycled aggregate and eliminating the finest particles, the possibility of such expansion decreases considerably. Regulation EHE-08 establishes the maximum content as 1% for total compounds containing sulphur [60].

Recycled aggregates do not present potential chemical reactivity with those alkalines present in concrete. In the case of recycled aggregates from one controlled source, that is concrete with known compositions and characteristics, instruction EHE-08 must be followed. To this end, a petrographic study was completed in order to obtain information on the type of reactivity that could occur. Although there are no limit values, it is essential that the recycled aggregates do not present any potential for reactivity.

When working with recycled materials, the presence of impurities and contaminants is commonplace. In recycled aggregates, such contaminants significantly and adversely affect the properties of ready-made concrete. Wood, plastic, gypsum, metal, glass, brick, asphalt, organic material, etc. are all common contaminants. One of the primary problems these contaminants pose for concrete is that they adversely affect its compressive strength. When the contaminating elements are clay or lime residues, compressive strength suffers even more than when recycled aggregate includes asphalt or paint waste. The type of contaminant found in recycled aggregates depends on the aggregate’s source. Recycled aggregate from concrete waste presents considerably fewer impurities than aggregates from demolition waste. EHE-08 establishes the limitations on contaminants and impurities as follows (Table 1).

The tests proposed herein verifies whether or not recycled aggregates comply with the requirements indicated by EHE-08 for their use and incorporation as a component of recycled structural concrete.

### 2.2. Characterization of Recycled Concrete

In the present study, coarse aggregate was incorporated as a replacement for natural aggregate without any additional modification in the concrete mix design, and maintaining the same quantity of cement and the same water-cement ratio. Recycled concrete was mixed using the same methods employed for traditional concrete.

A reference mix was prepared with natural aggregates. As noted above, the traditional aggregates for the concrete mix design came from the quarry La Peña, which is owned by the company Hormigones Ebro. In this case [61], three types of natural aggregates were utilized (regulatory names included): sand FA-0/6-T-S-L, and coarse aggregates GR-6/12-T-S-L and GR-12/20-T-S-L. The recycled aggregates utilized herein came from two different plants. The substitution percentages were realized only for the coarse fraction (12/20). The recycled aggregates were derived from: HE aggregates from concrete block waste, called CA-12/20-T-R; and HR aggregate derived from rejected materials when artificial aggregate is made from demolition waste, called CA-12/20-T-R. The cement utilized is 32.5 R, as this is the cement traditionally used to manufacture conventional concrete.

#### 2.2.1. Concrete Mix Design

Our present study focuses on concrete’s compressive strength as the primary data, based on the concrete mix design previously validated through characterization testing conducted at the ready-mix concrete plant. Table 2 displays the concrete mix design obtained for 1 m^3^. Since mixing is done in a laboratory with a smaller-sized mixer, the values presented are for 60 L of sample concrete, henceforth referred to as H0.

Based on the real mixes used in pre-mixed concrete plants, and establishing the amount of cement, a percentage of the natural coarse aggregate was replaced by recycled coarse aggregate. The goal was to create economically and environmentally viable mixes [41,48]. Thus, the mix proportions are listed in Table 2 for the different substitutions rates of recycled aggregate for natural aggregate (gravel 12/20), in the following proportions 15%, 20%, 30%, and 50%. Since there are two sources of recycled aggregates, the source is identified using the initials HE for Hormigones Ebro and HR for Hormigones Rioja, before indicating the percentage of substitution. Thus, there are 10 different mix proportions: one for the sample and four for verification for each of the two waste sources.

#### 2.2.2. Concrete Manufacturing Process

To manufacture each different mix, the different fractions of aggregate utilized were weighed separately (See Table 2). A calibrated scale was used for this task (Model COBOS_20K60, COBOS, Barcelona, Spain); it has a resolution of 1 g and a tolerance of ±3 g. Then, using the same scale, the cement and water were weighed. The container used to weigh the water was dampened before taring it.

To create the mix, a laboratory mixer was used with a capacity of 60 L (Model PROETISA 100I, PROETI, Madrid, Spain). Before introducing the materials in the mixer, the inside of the drum was moistened with water. The raw materials were placed in the cement mixer in the following order:

1. CA-12/20-T-S-L, mixer rotated for 3 s to evenly distribute material. In the substitution mixes, the corresponding fraction of CA-12/20-T-R was added at the same time.
2. CA-6/12-T-S-L, mixer rotated for 3 s to evenly distribute material.
3. FA-0/6-T-S-L, mixer rotated for 3 s to completely mix materials.
4. Cement was added and evenly distributed manually; the mixer was then switched on and rotated for 2 s.
5. Half the total water quantity was added and mixing resumed.
6. The remaining quantity of water was added with the mixer in motion during a period of time no greater than 30 s. This first mixing stage lasted 3 min and 30 s.
7. Let mix stand for 3 min with the mixer cover open.
8. After two minutes the mixing process was finished. Then ambient temperature and humidity were recorded, as well as the temperature of the cement mix.

Then, testing of the fresh concrete began and cylindrical specimens were prepared to test the hardened concrete. This mixing process was repeated three times for each different mix of concrete, so as to obtain average values for the results.

#### 2.2.3. Concrete Characterization Tests

**Figure 3:**
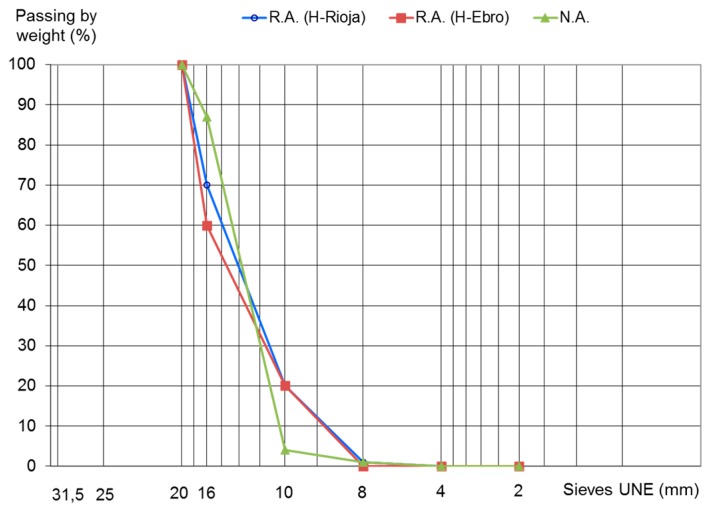
Grading of coarse aggregates utilized.

Various tests were performed on the concrete produced in each mix cycle (60 L): two consistency measurements with an Abrams cone in accordance with regulation UNE-EN 12350-2:2009 [62], occluded air according to regulation UNE-EN 12350-7:2010 [63] for fresh cement and, finally, seven cylindrical specimens (Figure 3) were obtained in compliance with UNE-EN 12390-2:2009/1M:2015 [64] to conduct the tests listed in Table 3.

The cylindrical specimens were faced with a polishing machine to reduce the dispersion in the results.

The cylindrical specimens were broken, by tensile force and compression, with an authorized and calibrated press (Model IBERTEST_MEH-3000-LCW, IBERTEST, Madrid, Spain). For the hardened concrete produced in this study, the following data was obtained for each mix: three measurements of compressive strength, aged seven days; nine measurements of compressive strength, aged 28 days; 12 measurements of density; nine measurements of absorption; nine measurements of indirect tensile strength; and nine measurements of water penetration.

Conducting the tests identified in Table 3 on hardened concrete provided information on the concrete’s performance. The aforementioned tests were conducted on concrete produced in the laboratory to determine the viability of incorporating recycled concrete aggregates.

## 3. Results and Discussion

This study analyzes the feasibility of obtaining structural concrete when recycled aggregates are utilized. The results are presented according to the characterization of recycled aggregates from structural concrete waste. This waste came from two different plants: Hormigones Ebro (HE), and Hormigones Rioja (HR). Natural aggregate is also characterized, which was replaced by recycled aggregate in different percentages. Table 4 lists the values for each of the different aggregates.

The results displayed in Table 4 confirm the suitability of recycled aggregates as substitutes for natural aggregates in the manufacture of recycled structural concrete. It is important to note that aggregate CA-12/20-T-R (HR) can only be employed to manufacture reinforced concrete given the high chloride content detected. After the tests were completed, the characterization of the grading of these aggregates was conducted. The results are shown in Figure 3.

Once the suitability of recycled aggregates as a substitute for natural aggregates in the manufacture of recycled reinforced concrete was verified, different mixes were made varying the percentage of coarse aggregate (CA) substituted and utilizing the mix proportions indicated in the methodology. The total number of mix designs is 10: that is, a total of five for each of the two different recycled aggregates from two different sources: a standard sample mix and four substitutions of coarse aggregate (15%, 20%, 30%, and 50%). The analysis of each mix was completed with three mixes used to test fresh and hardened concrete.

**Figure 4:**
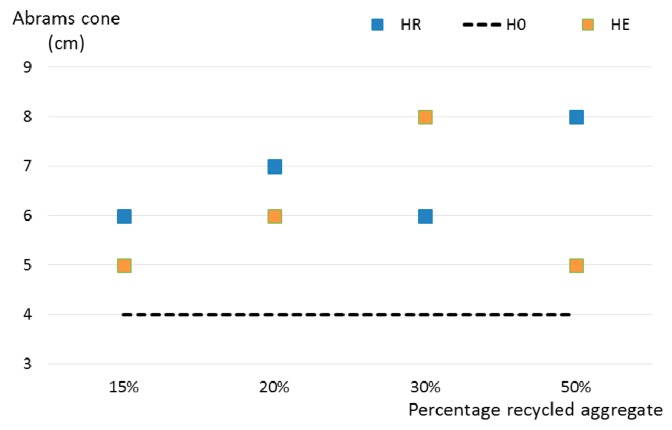
Consistency of manufactured concrete.

For fresh concrete, there is a generalized decrease in consistency (increased slump in an Abrams cone) when recycled aggregates are incorporated. However, the quantity of aggregate incorporated does not appear to influence the extent of said decrease. The data obtained is depicted graphically in Figure 4, wherein the results of the control concrete are indicated by a dashed line. The variations reflected in the graph often occur in concrete production. In this study, all the concrete, including recycled aggregates in the mix design, demonstrated concrete workability within the accepted regulatory range.

For the hardened concrete produced in this study, the following data was obtained for each mix. Table 5 presents a summary of the average values and standard deviations for each test of each of the mix proportions.

The results in Table 5 reveal that incorporating recycled aggregates leads to a slight decrease in concrete density. Regarding water absorption, including recycled aggregates increases the values, in general. This effect is more pronounced when the aggregate utilized is derived from ready-mix concrete waste (HE). The results of the water penetration test vary considerably and do not have a direct relationship with the quantity of substituted aggregate.

**Figure 5:**
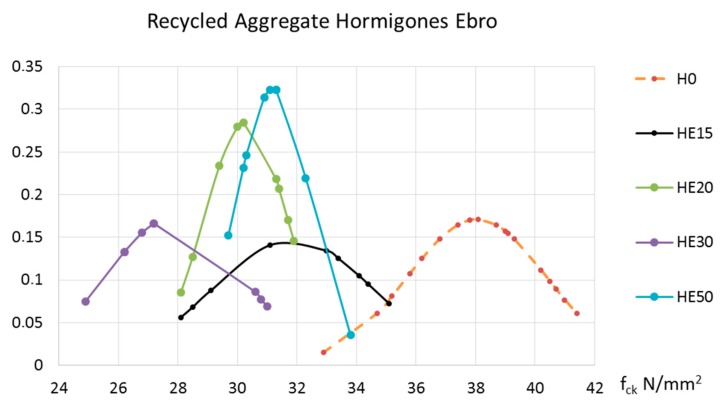
Compressive strength of concrete at an age of 28 days manufactured with recycled aggregates from Hormigones Ebro.

**Figure 6:**
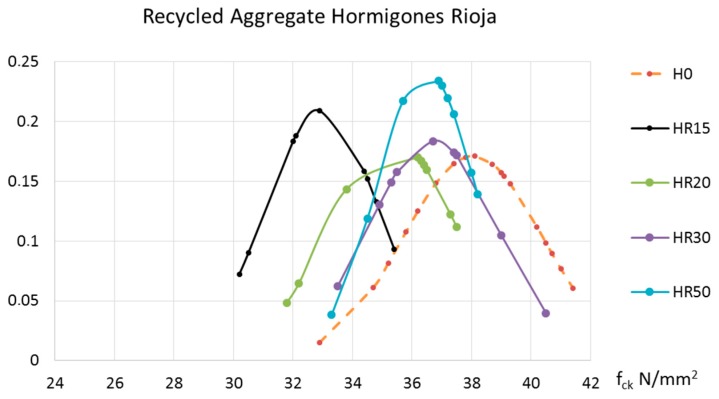
Compressive strength of concrete at an age of 28 days manufactured with recycled aggregates from Hormigones Rioja.

Compressive strength and indirect tensile strength have remarkable values for the performance of structural concrete. The tests realized after seven days aimed to follow-up and confirm that the cylindrical specimens were made correctly. The representative value for compressive strength at an age of 28 days (f_ck_) is one of the variables used to calculate structural concrete. In this case, using the data recorded, a normal distribution was carried out for each batch of concrete made. To facilitate the reader’s interpretation of the results, two graphs are included according to the source of the recycled aggregate. Figure 5 corresponds to the concrete manufactured with recycled aggregate from ready-mix concrete waste from Hormigones Ebro, and Figure 6 corresponds to the concrete made with aggregate from concrete demolition waste, treated at Hormigones Rioja.

Figure 5 and Figure 6 both contain the results of the control concrete manufactured with natural and H0 aggregates.

Including recycled aggregates from Hormigones Ebro had an adverse effect on the concrete’s compressive strength. This detrimental impact was even more pronounced in the case of the concrete from Hormigones Rioja.

**Figure 7:**
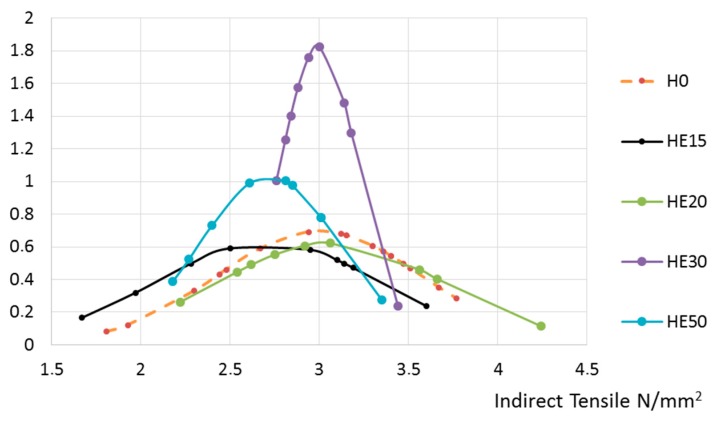
Concrete manufactured with recycled aggregates from Hormigones Ebro.

**Figure 8:**
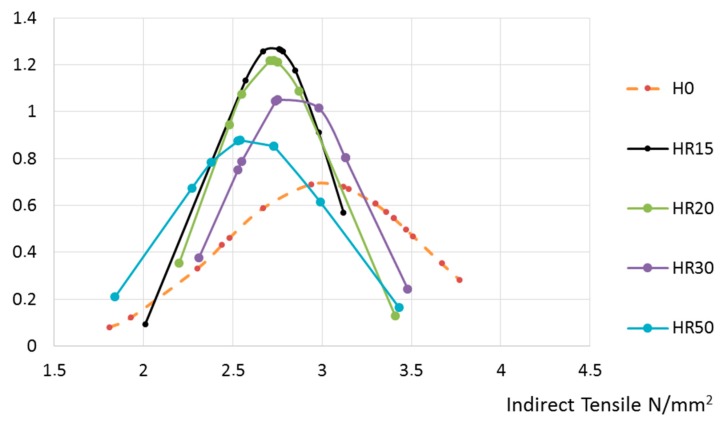
Concrete manufactured with recycled aggregates from Hormigones Rioja.

Then, the results obtained from the tensile tests were normalized. They are presented herein according to the recycled aggregate’s origin: Figure 7 for concrete made with aggregate from Hormigones Ebro, and Figure 8 for concrete made with aggregate from Hormigones Rioja.

Incorporating recycled aggregates from Hormigones Ebro (ready-mix concrete waste) or Hormigones Rioja (demolition waste) only slightly affected the indirect tensile strength, and the extent of this effect was not influenced by the amount of recycled aggregate used as a substitute.

Using recycled aggregates generated greater dispersion in the results. It was expected that increasing the amount of natural aggregate to be substituted would diminish the concrete’s strength properties. However, this phenomenon is not observed in the compressive strength of any of the cases examined herein. The dispersion present in the results is derived from the heterogeneous composition of the recycled aggregates. The source HE includes waste from pre-mixed concrete created in the plant: blinding concrete, non-structural concrete, and structural concrete. The HR source, on the other hand, consists exclusively of structural concrete from demolition waste. Thus, dispersion can be avoided by adequately managing and classifying said materials. The selection and classification of waste prior to producing recycled aggregate should be promoted and improved. Conducting this type of work beforehand improves the quality of the recycled aggregate produced. The use of recycled aggregate ought to be stimulated, given that its role will certainly take on increasing importance in the future. As the European Commission’s proposals recommend, it is important to plan for, and promote, the re-use of waste in the area where it is generated.

Spanish regulations recommend limiting recycled coarse aggregate to 20% of the total weight of materials used. This study demonstrates that a greater percentage of recycled aggregate can be incorporated if the materials to be recycled are characterized and treated before being processed. Economic and environmental limitations must be taken into consideration to recycle responsibly and sustainably. The results indicate that to produce non-structural concrete, the percentage of recycled aggregate used in the concrete mix can be increased.

## 4. Conclusions

This study demonstrates that the treatment of concrete through crushing and screening yields recycled aggregate that is suitable for producing structural concrete. In the case examined herein, the concrete manufacturing plants create the waste that is reincorporated into their product: this serves as a clear, practical, and effective example of a circular economy. Two sources of waste were analyzed.

In the case of recycled aggregates from Hormigones Rioja (demolition waste):

- The two treatments realized on the demolition waste entail significant costs. However, these treatments have been proven to contribute added value to the recycled aggregate obtained.
- The concrete produced with recycled aggregate at the HR plant obtained results similar to those of the control concrete.
- This study demonstrates that recycled aggregates that are made from concrete and undergo prior treatment can replace natural aggregate by up to 50% without provoking significant modifications in the produced concrete’s strength performance.

In the case of recycled aggregates from Hormigones Ebro (ready-mix concrete waste):

- There is a greater negative impact on the compressive strength of the concrete produced.
- The presence of low strength concrete in the HE source presents an obstacle for recycling with the aim of producing structural concrete.
- The concrete block aggregate was derived from concrete with a strength less than 25 N/mm^2^ at age 28 days. This type of waste, which undergoes a simple prior treatment of crushing and sieving, presents a significant quantity of adhered mortar.
- This undesirable effect can be remedied by introducing an initial classification step to categorize waste according to its strength. Thus, one could select only those recycled materials of equal or greater strength than the strength of the concrete to be made.

In the future, we should be able to avoid wasting materials when concrete is supplied to construction sites by efficiently commercializing the unused or leftover materials. Future lines of research should focus on concrete production processes with a reduced dispersion. Cataloging and classifying the materials to be used in concrete mixes must be an initial step to incorporating recycled materials into concrete production.

## Figures and Tables

**Table 1:** Limitations on impurities in recycled aggregate.

Contaminants and Impurities	Maximum % of Total Sample Weight
Ceramic material	5
Lightweight particles	1
Asphalt	1
Glass, plastic, metal	1

**Table 2:** Concrete mix design conducted in laboratory.

Material	Denomination	kg/m^3^	kg/0.06 m^3^				
			H0	15%	20%	30%	50%
Cement	CEM I-32.5R	300.0	18	18	18	18	18
Natural Aggregates	FA-0/6-T-S-L	765.0	50	50	50	50	50
	CA-6/12-T-S-L	405.6	24	24	24	24	24
	CA-12/20-T-S-L	601.5	36	30.6	28.8	25.2	18
Recycled Aggregates	CA-12/20-T-R			5.4	7.2	10.8	18
Water	Water supply network	163.0	9.78	9.78	9.78	9.78	9.78

**Table 3:** Tests conducted on cylindrical specimens.

Num. Cylindrical Specimens	Tests
1	Compressive strength, aged seven days [65].
2, 3, 4	Density [66], Absorption, and compressive strength, aged 28 days [65].
5, 6, 7	Density [66], indirect tensile strength [67] and water penetration [68]

**Table 4:** Characterization of natural and recycled coarse aggregates.

	CA-12/20-T-R (HE)	CA-12/20-T-R (HR)	CA-12/20-T-S-L (La Peña)	Limits EHE-08 [34]
% fine content	Free	0.10%	0.40%	<1.5%
Absorption	4.7%	3.3%	0.90%	RA < 20% Absorption < 7% (CA natural < 4.5%)
				RA > 20% Absorption < 5% (CA natural + CA recycled)
Clay lumps	0.08%	0.11%	Free	RA < 20% Clay Lumps < 0.6% (CA natural < 0.15%)
				Maximum amount 0.25%
Aggregate abrasion resistance	35%	24%	21%	<40%
Lightweight particles	Free	0.14%	Free	<1%
Determination of total chloride	0.015%	0.039%	0.006	Reinforced Concrete CA < 0.05% Pre-stressed Concrete CA < 0.03%
Alkali-aggregate reactivity	Aggregate not reactive	Aggregate not reactive	Aggregate not reactive	Recycled aggregates will not be reactive
Weight loss % magnesium sulphate	8.20%	1.00%	0.30%	<18% (CA)
Determination of sulphur compound	0.49	0.20	<0.10%	<1%
Determination of acid-soluble sulfates	0.28%	0.25%	0.02%	<0.80%

Recycled aggregate (RA); Coarse aggregate (CA).

**Table 5:** Results of the tests conducted on hardened concrete.

RF	Tests	H0		15%		20%		30%		50%	
		M	SD	M	SD	M	SD	M	SD	M	SD
HR	f_ck_ 7 days (N/mm^2^)	27.17	3.55	27.53	2.06	26.87	2.66	28.33	1.55	28.90	1.22
	f_ck_ 28 days (N/mm^2^)	36.54	2.27	32.98	1.91	35.33	2.16	36.70	2.17	36.47	1.65
	Density (g/cm^3^)	2.36	0.02	2.31	0.02	2.34	0.02	2.35	0.02	2.33	0.02
	Absorption (%)	2.09	0.12	2.45	0.30	2.12	0.28	2.09	0.20	2.11	0.17
	Indirect tensile (N/mm^2^)	3.23	0.44	2.72	0.31	2.71	0.33	2.84	0.37	2.60	0.45
	Water penetration (mm)	25.89	4.81	26.22	3.35	24.78	4.89	23.89	4.08	27.22	2.49
HE	f_ck_ 7 days (N/mm^2^)	31.07	1.62	28.83	4.27	27.63	2.08	25.40	1.87	28.00	1.04
	f_ck_ 28 days (N/mm^2^)	39.54	1.43	31.87	2,72	30.28	1.40	27.88	2.30	31.21	1.23
	Density (g/cm^3^)	2.38	0.02	2.37	0.02	2.36	0.02	2.34	0.04	2.33	0.02
	Absorption (%)	1.20	0.24	1.34	0.23	1.44	0.30	1.66	0.35	1.89	0.29
	Indirect tensile (N/mm^2^)	2.77	0.66	2.71	0.64	3.06	0.64	3.00	0.22	2.72	0.39
	Water penetration (mm)	29.67	5.36	34.56	12.13	30.00	2.18	28.44	3.28	26.67	2.50
